# Chemically induced cone degeneration in the 13-lined ground squirrel

**DOI:** 10.1017/S0952523824000014

**Published:** 2024-05-10

**Authors:** Hannah M. Follett, Emma Warr, Jenna Grieshop, Ching Tzu Yu, Mina Gaffney, Owen R. Bowie, Jong Won Lee, Sergey Tarima, Dana K. Merriman, Joseph Carroll

**Affiliations:** 1 Department of Cell Biology, Neurobiology, & Anatomy, Medical College of Wisconsin, Milwaukee, Wisconsin, USA; 2 Department of Ophthalmology & Visual Sciences, Medical College of Wisconsin, Milwaukee, Wisconsin, USA; 3 Joint Department of Biomedical Engineering, Marquette University and Medical College of Wisconsin, Milwaukee, Wisconsin, USA; 4 School of Medicine, Medical College of Wisconsin, Milwaukee, Wisconsin, USA; 5 Division of Biostatistics, Institute for Health and Equity, Medical College of Wisconsin, Milwaukee, Wisconsin, USA; 6 Department of Biology, University of Wisconsin Oshkosh, Oshkosh, Wisconsin, USA

**Keywords:** adaptive optics, cone-dominant, retinal degeneration, 13-lined ground squirrel, photoreceptors, retinal imaging

## Abstract

Animal models of retinal degeneration are critical for understanding disease and testing potential therapies. Inducing degeneration commonly involves the administration of chemicals that kill photoreceptors by disrupting metabolic pathways, signaling pathways, or protein synthesis. While chemically induced degeneration has been demonstrated in a variety of animals (mice, rats, rabbits, felines, 13-lined ground squirrels (13-LGS), pigs, chicks), few studies have used noninvasive high-resolution retinal imaging to monitor the *in vivo* cellular effects. Here, we used longitudinal scanning light ophthalmoscopy (SLO), optical coherence tomography, and adaptive optics SLO imaging in the euthermic, cone-dominant 13-LGS (46 animals, 52 eyes) to examine retinal structure following intravitreal injections of chemicals, which were previously shown to induce photoreceptor degeneration, throughout the active season of 2019 and 2020. We found that iodoacetic acid induced severe pan-retinal damage in all but one eye, which received the lowest concentration. While sodium nitroprusside successfully induced degeneration of the outer retinal layers, the results were variable, and damage was also observed in 50% of contralateral control eyes. Adenosine triphosphate and tunicamycin induced outer retinal specific damage with varying results, while eyes injected with thapsigargin did not show signs of degeneration. Given the variability of damage we observed, follow-up studies examining the possible physiological origins of this variability are critical. These additional studies should further advance the utility of chemically induced photoreceptor degeneration models in the cone-dominant 13-LGS.

## Introduction

The 13-lined ground squirrel (13-LGS) is a long-standing animal model used in vision research (Van Hooser & Nelson, 2006; Merriman et al., 2016). Features such as a cone-dominant retina, emmetropic eyes with a lens:globe ratio similar to humans, highly visual behaviors, expanded visual cortices, and amenability to noninvasive high-resolution imaging contribute to the utility of the 13-LGS as a model for vision research (Remé & Young, 1977; Gur & Sivak, 1979; Chou & Cullen, 1984; Sussman et al., 2011; Merriman et al., 2016; Sajdak et al., 2016). Furthermore, the 13-LGS is an obligate hibernator that demonstrates seasonal retinal remodeling with cone disruption (e.g., mitochondrial remodeling and cone outer segment (OS) shortening) observed during torpor followed by rapid recovery upon arousal (Remé & Young, 1977; Merriman et al., 2016; Sajdak et al., 2018; Sajdak et al., 2019; Zhang et al., 2020). Additional physiological adaptations, such as tolerance of brain hypoxia/ischemia and an ability to overcome metabolic stress during hibernation, enable a degree of neuroprotection that may extend to the retina (Boyer & Barnes, 1999; Ballinger et al., 2017). Finally, the sequenced 13-LGS genome and recent development of 13-LGS-induced pluripotent stem cells represent important achievements that should facilitate the advancement of the 13-LGS as a model for vision research (Ou et al., 2018; Grabek et al., 2019). Thus, there is great interest in developing robust and accessible retinal disease models using the 13-LGS.

One approach to inducing photoreceptor damage is retinal detachment, which has been demonstrated in a variety of animals, including pigs, rabbits, felines, and the cone-dominant chick retina, with variable results (Lewis et al., 2002; Cebulla et al., 2012). While rabbits exhibit widespread degeneration and cell loss, detachment studies in other species showed disrupted and damaged photoreceptors but limited cell death (Mervin et al., 1999; Faude et al., 2001). In feline experiments, reattachment of the retina results in a nonuniform recovery across the photoreceptor/retinal pigment epithelial interface as well as slow rod OS regrowth (Lewis et al., 2002). Experimental retinal detachment has also been used as a model of cone degeneration in the 13-LGS and the California ground squirrel, resulting in severe cone degeneration in both species (Jacobs et al., 2002; Linberg et al., 2002; Sakai et al., 2003; Salmon et al., 2018). The loss of cones corresponded to decreased electroretinogram amplitudes ranging from a 20% to 40% reduction to a near 100% reduction in larger detachments (Jacobs et al., 2002). However, subretinal injections can be challenging depending on ocular biometry, and the induced cone degeneration is limited to the detachment zone. As such, alternative models of cone degeneration are desired.

Chemically induced photoreceptor degeneration has been explored in a wide range of animal models, including the 13-LGS. For example, systemically administered iodoacetic acid (IAA) has been shown to produce photoreceptor degeneration in 13-LGS, beginning with widespread pyknotic nuclei and shortened outer and inner segments (ISs) by 3 days, near-total loss of OSs by 4 days, and reduction to masses of debris by 10 days (Farber et al., 1983). IAA has also been shown to result in degeneration of outer retinal layers in mice, swine, rabbits, felines, and non-human primates, but can be accompanied by thinning of other retinal layers and severe systemic effects (Noell, 1952; Scott et al., 2011; Rösch et al., 2015). Tunicamycin (Tm), an antibiotic known to inhibit protein glycosylation and induce stress in the endoplasmic reticulum, has been effective in producing outer retinal damage in both 13-LGS and California ground squirrels (Anderson et al., 1988). Tm has also been used in rats, mice, and guinea pigs to induce outer retinal degeneration, although subsequent degeneration of retinal capillaries was observed in mice (Shirai et al., 2015; Kageyama et al., 2019; Wang et al., 2019; Spencer et al., 2020). Additional chemicals such as adenosine triphosphate (ATP), sodium nitroprusside (SNP), and thapsigargin (Tgn) have been used in a number of species to induce photoreceptor damage (Puthussery & Fletcher, 2009; Aplin et al., 2014; Li et al., 2018; Kageyama et al., 2019), but not the 13-LGS. Across these studies, there has been limited use of noninvasive high-resolution retinal imaging to examine retinal structure. Here, we sought to characterize the effects of various intravitreally administered chemicals on cone photoreceptor structure in the 13-LGS using scanning light ophthalmoscopy (SLO), optical coherence tomography (OCT), and adaptive optics SLO (AOSLO).

## Methods

### Animals

All experimental procedures described were approved by the Institutional Animal Care and Use Committee of the Medical College of Wisconsin (AUA00005654). A total of 46 euthermic 13-LGSs (*Ictidomys tridecemlineatus*) were used for this study. Thirty-two animals (18M, 14F) came from the University of Wisconsin Oshkosh Squirrel Colony; their age at use was 7.5 months on average (range = 2 months-1.25 years old). Two euthermic 13-LGS adults (1M, 1F) were trapped from the wild in southeast Wisconsin; while their age at use was uncertain, the animals were considered sexually mature adults of at least 1 year of age as they were captured in early May 2019, shortly after emergence from hibernation (mid-April) and before litters are typically born (mid/late May). Twelve animals (7M, 5F) were born in captivity to wild-caught mothers; their age at use was 9.4 months on average (range = 2 months-1.25 years old). For reference, 13-LGS are fully weaned by ~1.5 months old, begin their first hibernation at ~4 months old, are sexually mature at ~11 months old, and typically reproduce in captivity until death at about ~7 years old (Merriman et al., 2012).

Experiments were conducted during the 2019 and 2020 active seasons (i.e., all 13-LGS were euthermic), taking place between April and October of each year (Supplementary Tables 1 and 2). All 13-LGS were housed under southeast Wisconsin’s natural photoperiod adjusted biweekly, and all procedures and imaging were performed within the natural photoperiod hours. Animals of less than a year of age did not hibernate prior to use, and animals of 11 months or older were allowed to hibernate before use.

### Chemicals and intravitreal injections

Dimethyl sulfoxide (DMSO), IAA, Tgn, and Tm were obtained from Sigma-Aldrich (St. Louis, MO), and SNP, ATP, and phosphate-buffered saline (PBS) were obtained from Thermo Fisher Scientific (Waltham, MA). Tgn and Tm were dissolved in 100% DMSO to prepare a stock solution and further diluted with sterile 1X PBS to achieve the desired final concentration. IAA, SNP, and ATP were dissolved in sterile 1X PBS only. All solutions were prepared on the day of use and protected from light until the time of injection.

Animals were anesthetized via isoflurane inhalation (5% induction, 2–4% maintenance in 1 L/min oxygen flow) using a non-rebreathing system (VetEquip, Inc., Livermore, CA, USA). Designated eyes were dilated and cyclopleged with 2.5% phenylephrine hydrochloride and 1% tropicamide (Akorn, Inc., Lake Forest, IL, USA), and 0.5% proparacaine hydrochloride (Akorn, Inc., Lake Forest, IL, USA) was applied for local anesthetic. Ophthalmic betadine (5%, Alcon Laboratories Inc. Geneva, Switzerland) was then used to sterilize the eye and surrounding area, and eyelids were held open with a modified rodent or pediatric speculum. Intravitreal injections were performed under a surgical microscope (M691; Leica Microsystems, Wetzlar, Germany). During the procedure, Gonak drops (Akorn, Inc., Lake Forest, IL, USA) or Refresh Celluvisc drops (Allergan Pharmaceuticals, Dublin, Ireland) were used to maintain lubrication and visualize the retina to prevent accidental lens or retinal injury. A 31G needle attached to a 100 μL Hamilton syringe or a tuberculin syringe was inserted approximately 1 mm nasal to the limbus, and a range of predefined concentrations and volumes of SNP, ATP, IAA, Tgn, or Tm were then administered (Table 1, Supplementary Tables 1 and 2).

**Table 1.** Summary of animals/eyes and chemical injections

Chemical	Animals (*n*)	Chemical eyes (*n*)	Vehicle eyes (*n*)	Volume	Concentration
ATP	*n* = 3 (3M)	3	3	30 μL	0.066 M
	*n* = 3 (3F)	3	3	30 μL	0.132 M
	*n* = 3 (2M, 1F)	6	0	20 μL	0.379 M
	*n* = 1 (1M)	1	0	30 μL	0.528 M
	*n* = 5 (3M, 2F)	8	0	10 μL	0.723 M
SNP	*n* = 2 (1M, 1F)	2	2	30 μL	0.10 mM
	*n* = 2 (1M, 1F)	2	2	30 μL	0.25 mM
	*n* = 4 (1M, 3F)	4	4	30 μL	1.2 mM
	*n* = 4 (3M, 1F)	4	4	30 μL	1.5 mM
IAA	*n* = 1 (1F)	1	1	30 μL	0.5 mM
	*n* = 1 (1M)	1	1	30 μL	1.0 mM
	*n* = 1 (1M)	1	1	30 μL	3.2 mM
	*n* = 1 (1F)	1	1	30 μL	4.7 mM
	*n* = 1 (1F)	1	1	30 μL	7.4 mM
	*n* = 1 (1M)	1	1	30 μL	15.4 mM
Tgn	*n* = 2 (2M)	2	0	30 μL	20 nM
	*n* = 2 (2M)	2	0	10 μL	82 nM
	*n* = 2 (2F)	2	0	10 μL	164 nM
Tm	*n* = 2 (2M)	2	0	30 μL	0.25 μg/μL
	*n* = 2 (1M, 1F)	2	0	10 μL	1.5 μg/μL
	*n* = 2 (1M, 1F)	2	0	10 μL	2.5 μg/μL

Abbreviations: ATP, adenosine triphosphate; SNP, sodium nitroprusside; IAA, iodoacetic acid; Tgn, thapsigargin; Tm, tunicamycin; F, female; M, male.

A total of 6 animals received chemical injections in both eyes, whereas the remaining 40 animals received chemical injections in only one eye. Of the latter, the contralateral eyes of 24 animals received an equal volume of 1X PBS to serve as an internal vehicle control. The contralateral eye of the remaining 16 animals was left uninjected to serve as an internal “wild-type” control to assess for possible contralateral effects. All 13-LGS recovered uneventfully from these injection procedures.

### Anesthesia and preparation for noninvasive retinal imaging

Prior to imaging, animals were anesthetized, dilated, and cyclopleged as described above. Animals were then placed in an imaging cassette affixed to an alignment stage capable of linear travel in the *x*, *y*, and *z* dimensions and rotational movement around a nodal point at which the eye was roughly aligned for imaging. During imaging, eyelids were held open with a modified rodent or pediatric ocular speculum, and wetting drops (Refresh Drops; Allergan Pharmaceuticals, Dublin, Ireland) were applied as needed throughout the experiments to maintain corneal hydration and a uniform tear film.

### Scanning light ophthalmoscopy

Baseline near-infrared (NIR; 820.5 nm) reflectance images were captured prior to chemical injections in all animals using a custom multiline confocal SLO (Spectralis HRA; Heidelberg Engineering, Heidelberg, Germany) and the widefield lens (55° widefield objective). In some animals (see Supplementary Table 2), follow-up NIR reflectance imaging was performed to screen for gross retinal changes, and short-wavelength autofluorescence (SW-AF; 486 nm excitation, 502–537 nm emission) imaging was performed to assess possible retinal pigmented epithelium (RPE) changes. Automatic real-time tracking in the Spectralis software (ver. 6.5.2.0) was used to register and average 20–50 frames for NIR reflectance images and 80–100 frames for SW-AF images.

### Optical coherence tomography

Baseline OCT was performed prior to chemical injections, and follow-up imaging was performed at 1, 3, 7, 14, and 21 days, or at 1, 2, 3, 4, 6, and 8 weeks (when possible due to alternate study; see Supplementary Tables 1 and 2) (Yu et al., 2024), to assess changes in retinal lamination. Retinal imaging was performed with a Bioptigen Envisu R2200 spectral domain OCT system (Leica Microsystems, Wetzlar, Germany) equipped with a superluminescent diode (SLD) (center wavelength 878.4 nm, 186.3 nm bandwidth) light source (Superlum, Cork, Ireland), using their Gen3 rabbit lens (90-BORE-G3-RB). One or more nominal 8 mm × 8 mm vertical volume scans of the retina (650 A-scans/B-scan, 300 B-scans) were acquired during each imaging session.

For each imaging session, a B-scan was extracted from the volume scans collected in each eye. To ensure selected B-scan frames were from approximately the same location across imaging sessions, OCT *en face* images were used to identify retinal landmarks and common locations across timepoints. *En face* images were automatically generated via summed volume projection during image acquisition in Bioptigen InVivoVue software or created from superficial capillary plexus segmentation slabs generated in a custom segmentation software (Scoles et al., 2016). Raw single B-scans from a common location in the central retina were identified in the longitudinal *en face* images by determining the number of *en face* image pixels across which a single frame in the image stack covered. The raw single B-scans were then extracted from the volume scans using ImageJ (NIH, Bethesda, MD), with the exception of three scans where poor image quality necessitated registration and averaging of up to three adjacent B-scans (Schneider et al., 2012). In these instances, the ImageJ TurboReg plugin was used to register and average the B-scans (Thévenaz et al., 1998), increasing the image signal-to-noise ratio without integrating across too large of a spatial range. Single B-scan frames or averaged B-scans were then cropped for subsequent analysis (50 pixels above the inner limiting membrane (ILM), 50 pixels below choroid). A total of 372 B-scans were extracted.

Longitudinal changes in retinal and choroidal thickness were assessed by segmenting the cropped B-scan images using DOCTRAP (Chiu et al., 2010; Chui et al., 2015). The extracted B-scans were masked and semiautomatically segmented by two observers. To assess interobserver agreement, a randomized 10% subset of images from each chemical group was segmented by both individuals. The remaining images were evenly distributed between observers by random assignment. Retinal OCT B-scans were semiautomatically segmented at the ILM, outer plexiform layer, RPE/Bruch’s membrane complex (RPE/BrM), and choroidal boundaries. Boundaries were manually adjusted as necessary to correct for segmentation errors. Total retinal and choroidal thicknesses were obtained by determining the axial distance (in pixels) between the ILM-RPE/BrM and RPE/BrM choroid, respectively, and converting to μm.

To define retinal eccentricities, first the transverse scale (*x*) of the OCT images (in degrees per pixel) was calculated using the following equation:

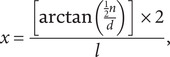

where *n* is the nominal scan length in mm, *d* is the assumed posterior nodal distance of the probe lens in mm, and *l* is the scan length in pixels.

Retinal eccentricity was then defined with respect to the center of the optic nerve head (ONH), which was identified as the area where the choroid was entirely absent (i.e., where the RPE/BrM and choroidal boundaries converged). Using a custom MATLAB script (MathWorks, Natick, MA; https://github.com/AOIPLab/OCT_Thickness_Extractor/releases/tag/Follett_et_al_2023), thickness values were then extracted and averaged from regions 0.75° wide, sampling from the ONH to 15.75° inferior of the ONH.

### Adaptive optics scanning light ophthalmoscopy

Baseline AOSLO imaging was performed prior to chemical injections, and at 7, 14, and 21 days, or at 4 and 8 weeks (when possible due to alternate study; see Supplementary Tables 1 and 2) to assess changes in the cone mosaic. Confocal and non-confocal videos were acquired simultaneously using a previously described custom AOSLO modified for a 4.5-mm system pupil diameter, and split detection and dark-field images were derived from the non-confocal videos as previously described (Dubra & Sulai, 2011; Sajdak et al., 2016). Imaging was performed with a 794-nm SLD (bandwidth = 15.1 nm; Superlum), an 845-nm (bandwidth = 26.9 nm; Superlum) wavefront sensing source, and either a 30 or 100 μm pinhole in the confocal detection channel. Aberrations of the wavefront were measured using a Shack–Hartmann wavefront sensor and corrected by a 97-actuator ALPAO deformable mirror with a 7.5 mm diameter (ALPAO; Montbonnot-Saint-Martin, France). Image sequences were first collected along the horizontal (ONH) to facilitate longitudinal imaging at the same location, followed by a 7–10° long inferior strip adjacent to a blood vessel. Up to 3 separate locations per eye were imaged with this protocol.

The scanning system’s inherent sinusoidal distortion was estimated by imaging a Ronchi grating with 118.1 lines/mm and corrected by resampling video frames over a grid of equally spaced pixels. The retinal image scale (*y*) in degrees per pixel was estimated using the following equation:

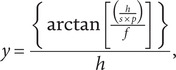

where *h* is the image height in pixels, *s* is the spacing between the lines of the Ronchi grating in lines/mm, *p* is the fringe period – that is, the number of pixels per line of the Ronchi grating (which varies depending on the scan angle of the specific image), and *f* is the focal length of the model eye (19 mm).

Frames in each collected image sequence were divided into strips and registered to reference frames automatically selected using a custom MATLAB script (Dubra & Harvey, 2010; Salmon et al., 2017). Strips with the greatest normalized cross-correlation values from 30 to 50 frames were then averaged to improve signal-to-noise ratio and create the final processed image. The resulting images were automatically montaged with a Scale Invariant Feature Transform (SIFT) algorithm and scaled to the smallest field-of-view (Chen et al., 2016). These automatically generated montages were transferred into Photoshop CS6 and inspected for accuracy with any gaps or misplaced images within the montage manually corrected as needed.

### Statistical methods

Interobserver agreement of OCT thickness measurements at each eccentricity was assessed using Bland–Altman analysis. Measurements within 0.75° inferior of the ONH were excluded from further analysis due to poor agreement. The effect of eccentricity, observer, and chemical group on log transformed choroidal thickness in chemical- and vehicle-injected eyes was modeled using linear mixed models (LMMs) with a third-degree polynomial approximation. The effect of eccentricity, observer, and chemical group on log transformed total retinal thickness in vehicle-injected eyes was modeled using LMM with a second-degree polynomial approximation. Clustering of observations within images was controlled by image random effect. This was followed by a backward variable selection to identify the parsimonious model. A significant observer effect was observed in choroidal thickness measurements and was accounted for in the regression models. Wald tests with 3 degrees of freedom were used to determine the statistical significance of treatment effects over time summarized by cubic polynomials. Post hoc statistical significance of regression coefficients (e.g., slopes, curvatures, and cubic terms of overtime trends) was also determined with Wald tests. Principal component analyses (PCAs) were used to identify principal components when the overall statistical significance of overtime trends could not be explained by the statistical significance of the regression coefficients due to multicollinearity. Statistical significance was determined by p < 0.05. Statistical modeling was done in R ver. 4.2.1 in a Windows 10 x64 environment using the *geepack* package ver.1.3.9 (Højsgaard et al., 2006). This package produced consistent estimates of covariance matrices, which were used for performing Wald tests.

## Results

### Iodoacetic acid

**Figure 1.**
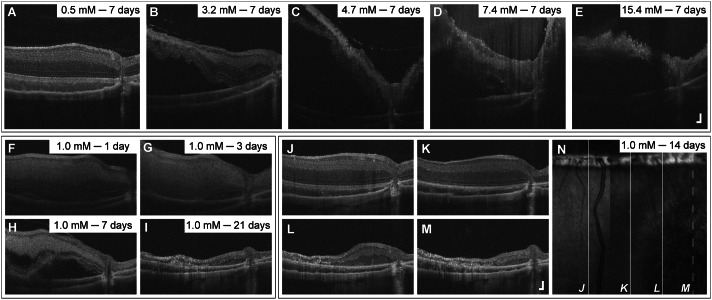
IAA induced pan-retinal damage that varied with concentration, time, and retinal location, as observed with OCT in five of six IAA-injected eyes administered different concentrations. While the eye that received the lowest concentration remained undamaged (A), retinal lamination in the remaining five eyes was severely disrupted and became detached. Images from damaged eyes at 7 days postinjection (B–E, H) demonstrate varying states of deterioration ranging from partially intact and attached lamination and detached regions with partially intact but degenerating lamination to complete deterioration of retinal integrity and full detachments. IAA-induced degeneration was also progressive. By 1 day postinjection, disruption of lamination was apparent and discrimination between retinal layers was limited (F). By 3 days, distinction between retinal layers was not possible (G), with detachments observed by 7 days (H) in all damaged eyes. Follow-up imaging in the 1.0-mM-injected eye showed a total loss of lamination at the 14- (M) and 21- (I) day postinjection timepoints. Damage from IAA was not uniform within damaged eyes. Outside of damaged regions, retinal lamination appeared normal (J) but became increasingly disrupted (K, L) until all lamination was lost within the lesion (M). The *en face* image (N) lines labeled *J-L* indicate the respective locations of J–L, and dashed line *M* indicates the location of F–I and M. OCT axial scale bar = 75 μm, lateral scale bar = 1°; lower right corner of panels E, M.

Injections of IAA were administered to a single eye in each of the six animals. The effect of IAA varied across concentrations. In the eye injected with the lowest concentration (0.5 mM), retinal lamination appeared normal on OCT (Fig. 1A), and AOSLO showed a contiguous cone mosaic. The remaining five IAA-injected eyes developed severe disruption of retinal lamination, resulting in detachment, as observed by OCT (Fig. 1B–E, H). IAA-induced damage also varied over time; diffuse reflectivity of retinal lamination and poor distinction between retinal layers was observed by 1-day postinjection (Fig. 1F) in all five damaged eyes. Damage progressed over the course of follow-up imaging. Deterioration of retinal lamination integrity and loss of distinction between retinal layers was observed by 3 days (Fig. 1G) in all five damaged eyes; retinal detachments were also observed by 3 days in the 15.4- and 7.4-mM-injected eyes and by 7 days in the 4.7-, 3.2-, and 1.0-mM-injected eyes (Fig. 1H). In only the damaged eye with follow-up imaging through 21 days postinjection in the same location, OCT showed severe disruption of retinal lamination resulting in loss of all layers (Fig. 1I); however, damage in this eye, as well as the other damaged eyes, varied with retinal location. Areas of relatively preserved lamination were observed with OCT in at least one timepoint in each damaged eye. As demonstrated in the 1.0-mM-injected eye, areas outside of the lesion appeared to have normal lamination on OCT (Fig. 1J, N, line *J*), which became progressively disrupted until it was lost entirely (Fig. 1K–M, N, lines *K–M*). AOSLO in this eye showed no remaining cone structure within the damaged area. All six contralateral eyes injected with PBS vehicle as negative controls showed no disruption in retinal lamination or cone mosaic contiguity.

### Adenosine triphosphate

ATP injections were administered to both eyes of six animals and to a single eye of nine animals (Supplementary Tables 1 and 2). Of the nine animals with a single ATP-injected eye, six received injections of PBS vehicle in the contralateral eye to serve as a vehicle control; the contralateral eyes of the remaining three animals stayed uninjected.

Up to 21 days postinjection, the two lowest ATP concentrations of 0.066 and 0.132 M (Table 1) yielded no difference from the uninjected control eyes, or from the PBS vehicle-injected eyes in terms of retinal lamination (per OCT) and cone mosaic contiguity (per AOSLO).

**Figure 2.**
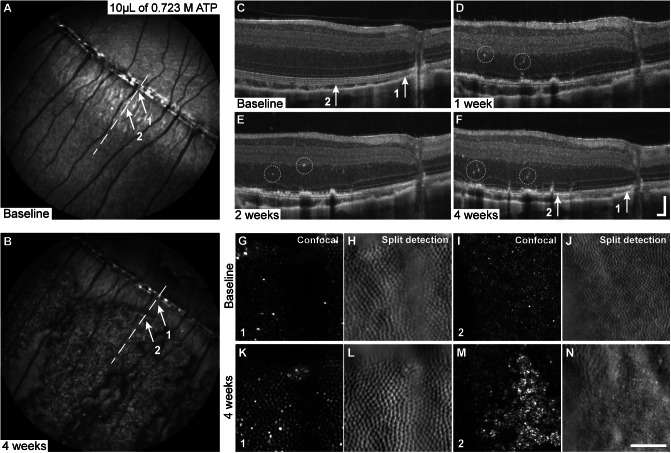
Altered NIR reflectance, disrupted outer retinal lamination, and disrupted cone mosaic regularity observed in eyes injected with high concentrations of ATP. Relative to baseline (A), patchy areas of mottled NIR reflectance bordered by reduced NIR signal appeared adjacent to normal-appearing fundus in the ATP-injected eyes with damage (B). Nonuniform disruptions to outer retinal lamination with hyper-reflective foci in the inner retina (dashed circles) were observed with OCT during weeks 1–4 weeks (C–F). On AOSLO, the cone mosaic appears contiguous at baseline (G–J) and at 4 weeks in areas with intact outer retinal lamination (location 1) (K, L) but disrupted in areas with altered lamination on OCT at 4 weeks (location 2) (M, N). Dashed lines on SLO denote location of OCT images. White arrows on the SLO and OCT images denote locations 1 and 2 of AOSLO images. OCT axial scale bar = 75 μm, lateral scale bar = 1°; lower right corner of panel F. AOSLO scale bar = 0.5°.

In contrast, the three highest ATP concentrations of 0.379, 0.528, and 0.723 M (Table 1) produced variable results. Of the 15 eyes injected, 10 eyes showed a range of disruptions in the outer retinal layers and cone mosaic. Damage appeared on NIR reflectance images as patchy areas of altered reflectance surrounded by areas of reduced reflectance at 1–4 weeks postinjection (Fig. 2B). Nonuniform disruptions in retinal lamination were visible with OCT at 1 week postinjection and progressed through 4 weeks (Fig. 2D–F). Within a single eye, areas of contiguous cone mosaic (Fig. 2K, L) and areas of patchy disruptions to the cone mosaic (Fig. 2M, N), including hyper-reflective debris and loss of IS structure, were visible with AOSLO at 4 weeks postinjection (Fig. 2G–N).

The variability in damage observed within individual retinas was also observed within and between concentrations. Eight eyes (*n* = 5 animals) received 10 μL injections of 0.723 M ATP; five of these eyes developed damage. Two of the damaged eyes were in animals that received bilateral ATP injections; however, damage was unilateral in these animals. One animal that received bilateral 10 μL injections of ATP also had damage in both eyes. Of the two animals with unilateral 10 μL ATP injections, only one developed damage. Six eyes (*n* = 3 animals) received 20 μL injections of 0.379 M ATP; damage was observed in only two eyes, both from the same animal. Damage was consistent between eyes and presented as disruptions on OCT and AOSLO, similar to the damage observed in the 10 μL eyes. Two eyes (*n* = 2 animals) received 30 μL injections of 0.528 M ATP; one eye was excluded because of technical error in the injection technique. Damage in the other eye was comparable to that observed in eyes with degeneration induced by 10 and 20 μL injections of high concentration (≥0.379 M) ATP. No effects were noted on the NIR reflectance images in any of the uninjected contralateral eyes.

### Sodium nitroprusside

**Figure 3.**
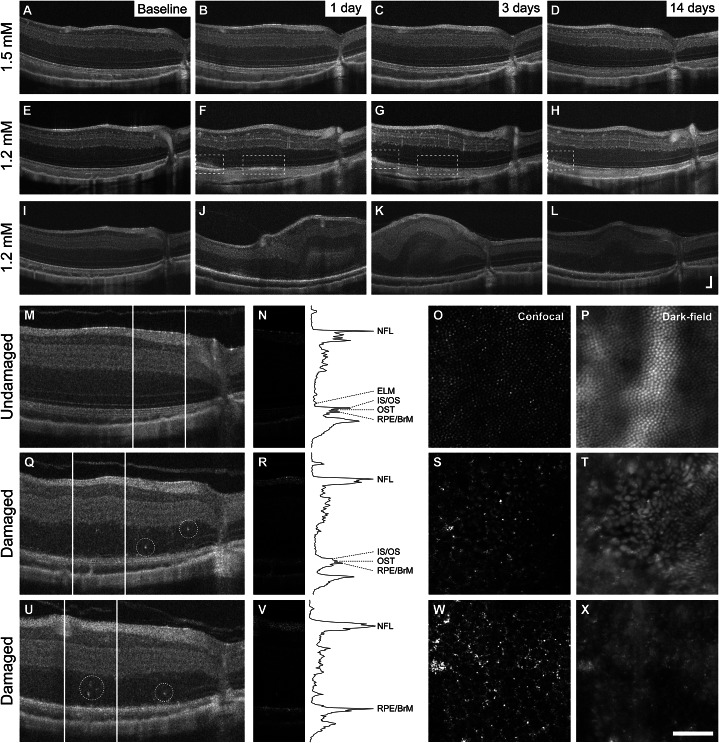
Variability in SNP-induced damage observed within chemical-injected eyes and between animals. Damage induced with higher concentrations (≥1.2 mM) of SNP was not observed in all animals receiving injections of equivalent or greater concentrations (a representative eye shown in A–D). When observed, damage induced by SNP presented on OCT as mild to severe distortion of retinal lamination by 1-day postinjection, resulting in nonuniform loss of outer retinal layers by 14 days. The eye shown in E–H and the eye shown in I-L were injected with the same volume and concentration of SNP. Dashed rectangles in E–H indicate areas of mildly distorted retinal lamination and resultant loss of outer retinal layers. Log OCT images from the 21 days postinjection timepoint of the eye in shown in I-L illustrate structure from an undamaged region (M) and variably damaged regions (Q, U) within the same retina. Hyper-reflective foci of unknown origin (dashed circle) could be observed in damaged regions. White lines on the log OCT images indicate where the linear-transformed OCT panels (N, R, V) were extracted from. The black lines plotted on the right are longitudinal reflectivity profiles (LRP) taken from the center of the linear OCT images, with peaks representing different retinal layers. Within each LRP, the top peak corresponds to the nerve fiber layer (NFL), and the bottom peaks correspond to outer retinal bands (ELM, external limiting membrane; IS/OS, inner segment/outer segment; OST, outer segment tip; RPE/BrM, retinal pigmented epithelium/Bruch’s membrane). AOSLO imaging in this damaged eye showed a contiguous cone mosaic (O, P) in undamaged areas where outer retinal bands were preserved on OCT (M, N), gross disruption of cone structure (S, T) in the damaged areas where outer retinal bands were still present but disrupted on OCT (Q, R), and complete loss of cone structure with visualization of the underlying RPE mosaic (W, X) within damaged areas where only the RPE/BrM band remained on OCT (U, V). OCT axial scale bar = 75 μm, lateral scale bar = 1°; lower right corner of panel L. AOSLO scale bar = 0.5°.

Injections of SNP were given to a single eye of each of the 12 animals, with their fellow eye receiving an injection of PBS vehicle (Table 1). Four animals received low concentrations of SNP (≤0.25 mM), which did not result in degeneration. The highest SNP concentrations of 1.2 and 1.5 mM produced variable degeneration of outer retinal layers in six of eight chemical-injected eyes, ranging from subtle to severe disruption of retinal lamination on OCT (Fig. 3). At 1 day postinjection, differentiation between outer retinal bands, including the external limiting membrane, IS/OS, OS tips (OST), and RPE/BrM, was impossible in areas with degeneration (Fig. 3F, J). SNP-induced distortion often impacted all retinal layers, although these observations were not observed uniformly across the retina. This distortion subsided by 7–14 days postinjection, leaving little to no outer retina behind in the most severe instances, while sparing the inner retina in these regions (Fig. 3H, L).

**Figure 4.**
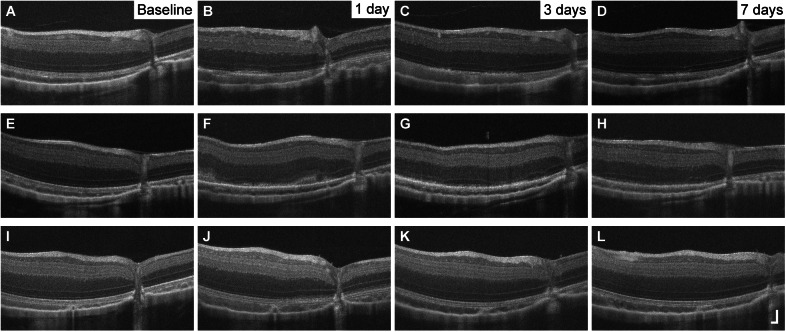
SNP-induced damage was observed in some contralateral vehicle-injected control eyes and varied between animals. OCT imaging showed mild to moderate disruption of retinal lamination was present by 1 day postinjection, resulting in a range of outcomes by 14 days including mild disruption of outer retinal layers (A–D; contralateral to a 1.2 mM injection), nonuniform loss of outer retinal layers (E–H; contralateral to a different 1.2 mM injection), or retained lamination (I–L; contralateral to a 1.5 mM injection). OCT axial scale bar = 75 μm, lateral scale bar = 1°; lower right corner of panel L.

In damaged eyes, patchy disruption of the cone mosaic was evident on AOSLO. In eyes with severe degeneration, locations with contiguous cone mosaic and intact outer retinal bands on OCT (Fig. 3M–P) could be observed in close proximity to areas of degeneration where debris and the underlying RPE mosaic were visible and outer retinal bands had been lost (Fig. 3U–X). In regions of damage between undamaged locations and damaged areas with RPE visibility, disruptions in the cone mosaic and the outer retinal bands were apparent (Fig. 3Q–T). On OCT, hyper-reflective foci of unknown origin were interspersed throughout damaged regions with and without disrupted outer retinal bands on OCT images, and gaps and abnormalities in the cone mosaic were visible on dark-field images in adjacent regions as well as otherwise normal-looking areas (not shown). Of the six animals with damage, four were also observed to have disruption in their fellow PBS vehicle-injected eyes (Fig. 4). The severity of contralateral degeneration was also variable, with one PBS-injected eye (Fig. 4E–H) showing damage of similar severity to the corresponding SNP-injected eye (Fig. 3I–X).

### Thapsigargin

**Figure 5.**
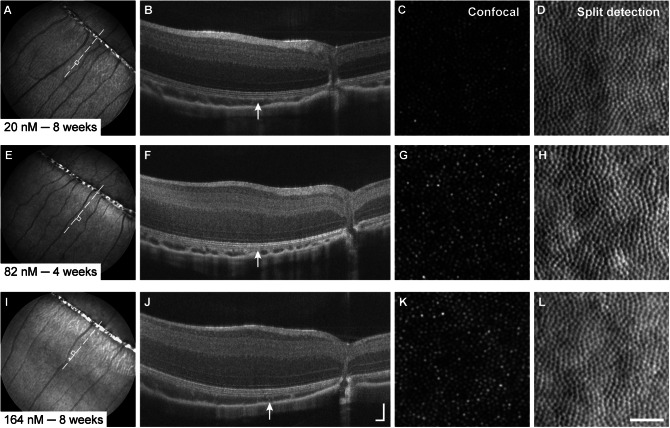
No degeneration was observed in thapsigargin-injected eyes at any concentration tested. In representative images from 4 or 8 weeks postinjection, NIR reflectance images of the fundus appeared normal (A, E, I); OCT revealed no apparent abnormalities in retinal lamination (B, F, J); and AOSLO revealed no apparent abnormalities in the cone mosaic (C, D; G, H; K, L). Dashed lines in NIR images denote locations of corresponding OCT images; white boxes/arrows denote locations of corresponding AOSLO images OCT axial scale bar = 75 μm, lateral scale bar = 1°; lower right corner of panel J. AOSLO scale bar = 0.25°.

Six animals received a single, unilateral injection of Tgn. Eyes injected with Tgn did not show any damage at any of the three concentrations tested. No abnormalities in NIR reflectance (Fig. 5A, E, I) or in SW-AF images were observed. No disruptions in retinal lamination were observed with OCT (Fig. 5B, F, J), and AOSLO images of the cone mosaic did not reveal any abnormalities (Fig. 5C, D, G, H, K, L). No changes in the appearance of the fundus of contralateral eyes were observed on NIR reflectance or SW-AF SLO images.

### Tunicamycin

**Figure 6.**
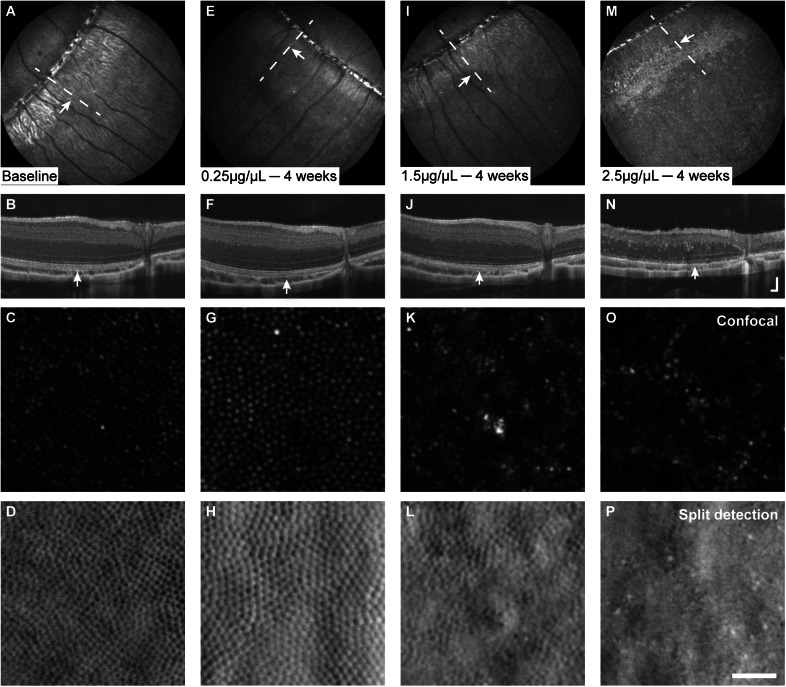
Disruption of retinal lamination and appearance of the cone mosaic varied with concentration of tunicamycin (Tm). Baseline images from 1.5 μg/μL animal provided for reference (A–D). The lowest concentration of Tm (0.25 μg/μL) did not produce degeneration detectable with SLO (E); OCT (F); or AOSLO (G, H). Higher concentrations (≥1.5 μg/μL) of Tm resulted in widespread alterations in NIR reflectance, retinal lamination, and cone mosaic appearance. In eyes injected with 1.5 μg/μL Tm, subtle, nonuniform disruptions in NIR reflectance and outer retinal lamination were observed on SLO (I) and OCT(J), with AOSLO showing altered reflectance of the cone mosaic on confocal images (K) and mostly retained inner segment structure on split detection images (L). Changes in NIR reflectance (M) and disruptions in lamination (N) were more severe in eyes receiving 2.5 μg/μL Tm, with AOSLO showing widespread loss of cone structure (O, P). Dashed line indicates location of OCT images, white arrows denote locations of AOSLO images. OCT axial scale bar = 75 μm, lateral scale bar = 1°; lower right corner of panel N. AOSLO scale bar = 0.25°.

Intravitreal injections of Tm were administered to one eye in each of the six animals. At all but the lowest concentration (0.25 μg/μL), Tm produced outer retina-specific degeneration (Fig. 6). In eyes injected with 1.5 μg/μL Tm, OCT images revealed subtle reflectivity changes of outer retinal layers and decreased distinction between IS/OS, OST, and RPE/BrM layers at 1 week postinjection. By 4 weeks postinjection, distinction between IS/OS, OST, and RPE/BrM bands on OCT was not possible, and AOSLO images revealed a discontiguous cone mosaic with hyper-reflective structures of unknown origin (Fig. 6I–L). Injections of 2.5 μg/μL Tm produced nonspecific damage leading to total retinal thinning from 2 to 4 weeks, as seen on OCT images (Fig. 6N). Anterior segment OCT scans also revealed lens opacifications and posterior synechia at 2 weeks, which progressively impeded OCT and SLO imaging and precluded 4-week follow-up AOSLO imaging in one eye. In the eye where AOSLO imaging was still possible at 4 weeks postinjection, hypo-reflective regions interspersed with hyper-reflective structures were observed in confocal images, with the absence of cone structure observed in split detection (Fig. 6O, P). NIR reflectance SLO images of the uninjected control eyes revealed no changes in fundus characteristics that would indicate contralateral damage.

### Assessments of retinal and choroidal thickness

**Figure 7.**
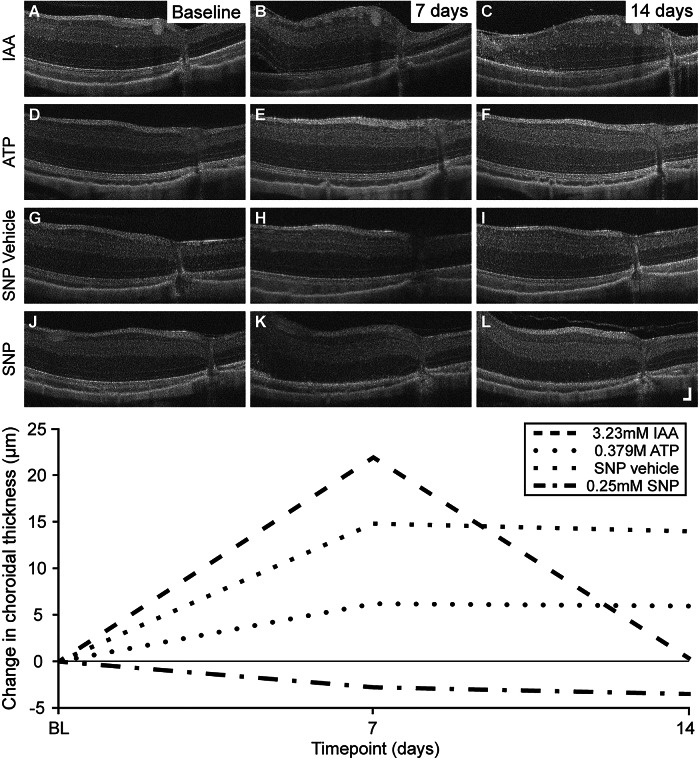
Transient changes in choroidal thickness observed on OCT in the week following injections. Some eyes showed transient increases in choroidal thickness within 1 week of injection, which resolved by 2–3 weeks. These choroidal thickness changes were observed most often in both chemical- and vehicle-injected eyes of animals receiving IAA (A–C; chemical-injected), ATP (D–F; chemical-injected), or SNP (G–I; vehicle-injected, contralateral to 0.25 mM SNP). Variability in amount and duration of choroidal thickness changes was observed between and within chemical groups, with some eyes demonstrating little to no change (J–K; SNP-injected eye). Each row of images is from a separate representative eye. Choroidal thickness normalized to the baseline average is plotted from each of these representative images. OCT axial scale bar = 75 μm, lateral scale bar = 1°; lower right corner of panel L.

Due to the severity of IAA-induced damage observed, image interpretation and alignment across timepoints was not always possible; thus, in eyes injected with the highest concentrations (≥3.23 mM) of IAA, only a subset of images from the earlier timepoints met the inclusion criteria for analysis. In all six animals injected with IAA, a transient increase in choroidal thickness was observed in both chemical- and vehicle-injected eyes (Fig. 7A–C). Thickness increases peaked between 1 and 3 days, followed by a variable reduction through 14 days. These changes were found to be significantly associated with the administration of IAA (Table 2; Supplementary Table 3). Changes in total retinal thickness in the vehicle-injected eyes were not found to be significantly different compared to other PBS vehicle control groups (Table 2).

**Table 2.** Impact of chemical and vehicle injections on choroidal and total retinal thickness based on linear mixed models

Variables with significant effects in parsimonious choroidal thickness model						
Variables	Slope		Curvature		Cubic term	
	*β*	*p*	*β*	*p*	*β*	*p*
ATP	0.07622	<0.001	−0.00789	0.0024	0.00021	0.0159
ATP vehicle	0.09485	<0.001	−0.01032	0.0022	0.00029	0.0093
IAA	0.19598	<0.001	−0.02099	<0.001	0.00058	<0.001
IAA vehicle	0.10501	<0.001	−0.01175	0.0018	0.00034	0.0058

Results of total retinal thickness model in vehicle-injected eyes					
Variables	Slope		Curvature		Overall *p*-value
	*β*	*p*	*β*	*p*	
ATP vehicle	0.00621	0.2081	−0.00033	0.1886	0.40
IAA vehicle	0.00119	0.5962	−0.00002	0.8396	0.09
SNP vehicle	0.00456	0.0137	−0.00020	0.0467	0.025

In the ATP group, 66.7% of chemical-injected eyes and all PBS vehicle-injected eyes showed a subtle to moderate increase in choroidal thickness following injections. The increase in thickness was transient, appearing as soon as 1-day postinjection and stabilizing to near normal measurements between 2 and 3 weeks postinjection (Fig. 7D–F). The observed transient changes in choroidal thickness were found to be significant in both PBS vehicle- and ATP-injected eyes (Table 2; Supplementary Table 3). The incidence of increased choroidal thickness at 1 week postinjection varied with injection volume, where an increase was observed in 54% of eyes injected with 30 μL (either ATP or PBS), 83% of 20-μL-injected eyes, and 62% of 10-μL-injected eyes. Measurements of retinal thickness in the vehicle-injected eyes did not significantly change compared to other PBS vehicle control groups (Table 2).

Following injections of SNP, an apparent transient thickening of the choroid in the week following injections was observed in 75% of chemical- and 83% of PBS-injected eyes (Fig. 7G–L). The increase in thickness peaked between 1 and 3 days and slowly decreased through 14–21 days. Changes in choroidal thickness were not found to be significantly associated with injections of SNP based on the initial 2D LMMs but were significant in the 3D Wald tests (Supplementary Table 3). Post hoc tests of slopes, curvatures, and cubic terms of overtime trends of the SNP- and contralateral vehicle-injected eyes were not significant. However, PCAs showed that the second and the third principal components of the overtime effects in the SNP- and contralateral vehicle-injected eyes produced *p*-values <0.05 (Supplementary Table 3). Changes in total retinal thickness in the contralateral vehicle-injected eyes over time were found to be significantly different compared to other PBS vehicle control groups (Table 2).

Assessments of choroidal thickness in Tm animals at 1 week postinjection showed a moderate increase in a 10 μL injected animal and a moderate decrease in a 10 μL injected animal. In the remaining four animals, the thickness measurements were relatively unchanged. Choroidal thickness fluctuated in all animals through 21 days postinjection. Similar findings were seen in the Tgn-injected group. A slight increase in choroidal thickness was observed in a 10 μL injected animal and a 30 μL injected animal, whereas measurements in the remaining four animals were relatively unchanged at 1 week postinjection. Choroidal thickness again varied through 21 days postinjection. None of the choroidal thickness changes in the Tm or Tgn groups were significant based on the LMMs and the 3D Wald tests (Supplementary Table 3).

## Discussion

In this study, we demonstrated chemically induced retinal degeneration in the 13-LGS detected using a suite of noninvasive imaging methods suitable for longitudinal study. There was substantial variation in response to different chemicals as well as within and between concentrations of the same chemical. While IAA produced severe pan-retinal damage at all but the lowest concentration and Tgn failed to induce damage at every concentration tested, most concentrations of SNP, ATP, and Tm created outer retina-specific degeneration. Transient increases in choroidal thickness were also observed in the IAA, ATP, and SNP groups, although fluctuation in thickness was present within all groups. Variation in the response to chemical administration and the extent of the damage have also been observed in other models of chemically induced degeneration, including IAA and ATP studies in felines (Aplin et al., 2014; Myeong Noh et al., 2019), IAA studies in 13-LGS (Farber et al., 1983), ATP studies in rats (Vessey et al., 2014), SNP studies in rabbits (Li et al., 2018), and Tm studies in guinea pigs and ground squirrels (California and 13-LGS) (Anderson et al., 1988; Spencer et al., 2020). While the variability was not always directly referenced, studies that did highlight variable observations speculate these discrepancies arose from a range of sources, such as differences in injection procedure, differences in intraretinal diffusion, and insensitive assessments (Anderson et al., 1988; Li et al., 2018). In our study, a possible source of variability stems from the fact that the 13-LGS is an obligate hibernator that demonstrates drastic physiological changes in preparation for and during hibernation (Remé & Young, 1977; Boyer & Barnes, 1999; Merriman et al., 2016; Ballinger et al., 2017). Chemical injections and follow-up imaging were performed throughout the active season of April–October; however, test-bouts of torpor can begin to occur as early as July, increasing in frequency until the hibernation season commences. If and to what extent neuroprotection and remodeling mechanisms are present during these test-bout events, and if/how much they influence or protect against the effect of a chemical to produce damage is unknown. It is also possible the strong circannual rhythm driving the hibernation cycle in the 13-LGS, as well as their diurnal nature, may have contributed to the variability in choroidal thickness data. Although circadian fluctuations in choroidal thickness, axial length, and intraocular pressure have been documented in humans and other species (Nickla et al., 2002; Nickla, 2006; Read et al., 2008; Usui et al., 2012), the influence of circadian and circannual changes on ocular biometry and choroidal thickness has not been investigated in the 13-LGS. Other factors such as slight misalignments between longitudinal OCT B-scans, differences in OCT B-scan retinal eccentricity between animals, the unquantifiable volume of solution lost to reflux, different chemical concentrations acting on the retina due to differences between animals in eye size/vitreous volume, and possible inconsistencies in injection techniques may also have contributed to the variability in our data. In addition, 13-LGS population variability is to be expected given the natural and outbred nature of this animal model. Nevertheless, our results demonstrate the feasibility of creating chemically induced damage specific to the cone photoreceptors in the 13-LGS.

Perhaps the most surprising result of the study was the degeneration observed in four of the eight PBS vehicle-injected eyes of animals injected with the highest two SNP concentrations (≥1.2 mM) in their contralateral eye. This damage ranged from subtle disruptions of outer retinal bands to severe disruption of outer retinal layers and loss of the outer nuclear layer (ONL), similar to that observed in the corresponding chemical-injected eye. Systemic effects from some ophthalmic drugs, such as topical β-blockers and intraocular injections of anti-VEGF, have been reported (Falavarjani & Nguyen, 2013). Interestingly, a recent gene therapy trial for Leber hereditary optic neuropathy showed contralateral improvement, further supporting a possible mechanism for the interocular transfer of physiological/therapeutic effects (Yu-Wai-Man et al., 2020). A partial explanation may lie in the arterial system. Multiple animal studies investigating vasculature found interophthalmic arteries present in rabbits and guinea pigs, providing a possible route for the transfer of drugs as well as materials produced in response to trauma (Forster et al., 1979; Kuchinka, 2017). While the mechanisms underlying these interocular effects are not fully understood, it is interesting that we only observed contralateral damage in the SNP animals. As no contralateral damage was observed in the ATP and IAA cohorts with PBS vehicle injections or in the ATP, Tm, and Tgn cohorts with uninjected fellow eyes, it is unlikely that the vehicle and/or injection procedure were responsible for the contralateral effect, thereby suggesting the effect was induced by SNP.

In addition to the contralateral effects, it was surprising that the chemical concentrations required to produce damage were often greater in 13-LGS than those reported in other species. Intravitreal injections of ATP in felines and rats induced apoptosis of photoreceptors through overactivation of P2X_7_ purinoceptors, a cell death pathway other retinal neurons are resistant to until higher extracellular ATP concentrations are reached (Puthussery & Fletcher, 2009; Aplin et al., 2014). In these studies, the concentrations used to induce photoreceptor damage (0.2 M) were lower than what was required here to induce similar damage in 13-LGS (0.379 M–0.723 M). SNP produced outer retinal damage in rabbits and chicks, and higher concentrations also produced a significant increase in pigment observed in the ONL of contralateral control eyes in chicks (Carr & Stell, 2016; Li et al., 2018). Damage-inducing concentrations in these species ranged from 10 nM to 0.5 mM, whereas the concentrations required to produce degeneration in 13-LGS ranged from 1.2 to 1.5 mM. Tunicamycin studies in rats, mice, guinea pigs, and ground squirrels produced outer retina-specific degeneration through both systemic and intravitreal delivery routes (Anderson et al., 1988; Kageyama et al., 2019; Wang et al., 2019; Spencer et al., 2020). In these studies, concentrations delivered via intraocular administration ranged from 0.025 μg/μL in mice (Wang et al., 2019) to 1 μg/μL in ground squirrels (Anderson et al., 1988) to produce outer retinal damage. In our study, however, mild outer retina-specific damage was observed at mid-range concentrations (1.5 μg/μL) and pan-retinal damage at higher concentrations (2.5 μg/μL). While the impact of the DMSO/PBS used to create solutions of Tm and Tgn was not directly assessed, a lack of damage in the eyes receiving the lowest concentration of Tm as well as the eyes injected with Tgn suggests that the vehicle did not contribute to the damage observed at higher Tm concentrations, although future studies should be done to validate this observation. Most studies on IAA-induced degeneration delivered the chemical through some means of systemic administration, which resulted in outer retinal damage as well as many side effects and a high mortality rate (Noell, 1952; Farber et al., 1983; Rösch et al., 2015). Conversely, we produced areas of pan-retinal damage via intravitreal injections. *In vivo* and *ex vivo* Tgn studies in rats showed selective degeneration of photoreceptors (Kageyama et al., 2019); however, none of the concentrations of Tgn tested here produced damage in the 13-LGS. While our study required higher chemical concentrations to induce photoreceptor damage in the 13-LGS, these results are not conclusive and further investigation into the underlying reasons is warranted.

There were numerous limitations to our study. A low number of animals for certain chemicals and concentrations may have prevented the identification of outliers, and a lack of correlative histology prohibits the confirmation of damage/cellular specificity speculated through image interpretation. In addition, quantitative analysis of degeneration was limited to OCT from a single location within a retina. Multiple p-values and the potential for overfitting of the modeling approach used for OCT analysis might contribute to an increased rate of false findings of significance for some results, such as the changes in total retinal thickness of vehicle-injected contralateral control eyes of SNP groups. Similarly, multicollinearity between estimates of the model’s regression coefficients has the potential to undermine potentially significant effects, such as the regression coefficients that reflected longitudinal changes in choroidal thickness of SNP-injected eyes and their fellow vehicle-injected eyes, as detected with the overall Wald tests and explained by the PCA. If these findings are indeed significant, determining what drives choroidal thickness changes in SNP animals and disambiguating whether the total retinal thickness changes observed in the contralateral PBS-injected eyes are due to direct effects of the vehicle or arise from interocular or systemic interactions requires further study. Furthermore, the size of the eye was ignored when calculating the chemical concentration. Given the impact of vitreous fluid dynamics on diffusion of a solution throughout the vitreous cavity, the concentration of the chemical at the retina is unknown. In addition, we did not assess or control for the hibernation-related physiological changes that animals undergo throughout the active season, and thus the extent to which our results were influenced is unclear. Finally, it was not possible to follow all animals at the exact same timepoints throughout the study due to inclusion in an alternate study (Yu et al., 2024) and revision of study design. Despite these limitations, the data show the creation of chemically induced photoreceptor degeneration as visualized through the use of *in vivo* retinal imaging.

We successfully produced outer retinal damage with chemicals in 13-LGS. While many studies use chemicals to induce photoreceptor degeneration, noninvasive monitoring of the progression and specificity of damage represents an important advance toward the translational use of models like these. Also, the use of noninvasive and high-resolution imaging technologies to monitor damage is advantageous as it reduces animal use and allows for comparison to findings using the same imaging tools in humans with retinal degenerative disease. Additional studies investigating the impact of seasonal changes and eye volume on damage creation are needed to further optimize this model. The ability to quickly and efficiently create a cone-dominant animal model of chemically induced photoreceptor degeneration has many potential applications in vision research, including use as a model in testing of therapeutics for vision restoration.

## Supporting information

Follett et al. supplementary material 1 Follett et al. supplementary material

Follett et al. supplementary material 2 Follett et al. supplementary material

Follett et al. supplementary material 3 Follett et al. supplementary material

## Supplementary material

The supplementary material for this article can be found at http://doi.org/10.1017/S0952523824000014.
