# Supplementary material for: Chemically induced cone degeneration in the 13-lined ground squirrel
Source: Vis Neurosci. 2024 May 10;41:E002. doi: 10.1017/S0952523824000014 (PMC11106521; doi:10.1017/S0952523824000014)
Supplement: Follett et al. supplementary material 1 — Follett et al. supplementary material [file S0952523824000014sup001.docx]

Supplemental Table 1: Animal demographics, treatments, imaging dates for 2019 experiments

| Supplemental Table 1: 2019 Experiments | | | | | | | | | | | |
| --- | --- | --- | --- | --- | --- | --- | --- | --- | --- | --- | --- |
| Animal ID | Sex | Age | Chemical | Volume & conc. | Baseline | Injection | 1 Day | 3 Days | 7 Days | 14 Days | 21 Days |
| DM_191301 | M | ~2.5 months | SNP | 30 µL 0.10 mM | OCT: 6/13  SLO: 6/12  AOSLO: 6/13 | 6/19 | OCT: 6/20 | OCT: 6/22 | OCT: 6/26 | OCT: 7/3 | OCT+AOSLO: 7/10 |
| DM_191206 | F | ~2.5 months | SNP | 30 µL 0.10 mM | OCT: 6/14  SLO: 6/17  AOSLO: 6/17 | 6/18 | OCT: 6/19 | OCT: 6/21 | OCT: 6/25 | OCT: 7/2 | ND |
| DM_191205 | M | ~2.5 months | SNP | 30 µL 0.25 mM | OCT: 6/14  SLO: 6/12  AOSLO: 6/17 | 6/18 | OCT: 6/19 | OCT: 6/21 | OCT: 6/25 | OCT: 7/2 | ND |
| DM_191208 | F | ~2.5 months | SNP | 30 µL 0.25 mM | OCT: 6/12  SLO: 6/12  AOSLO: 6/19 | 6/19 | OCT: 6/20 | OCT: 6/22 | OCT: 6/26 | OCT: 7/3 | OCT+AOSLO: 7/10 |
| DM_191209 | M | ~3.5 months | SNP | 30 µL 1.2 mM | OCT: 6/14  SLO: 6/12  AOSLO: 7/11 | 7/12 | OCT: 7/13 | OCT: 7/15 | OCT: 7/19 | ND | ND |
| DM_190906 | F | ~4 months | SNP | 30 µL 1.2 mM | OCT: 7/30  SLO: 7/30  AOSLO: 8/13 | 8/13 | OCT: 8/14 | OCT: 8/16 | OCT: 8/20 | OCT+AOSLO: 8/27 | ND |
| DM_190904 | F | ~4 months | SNP | 30 µL 1.2 mM | OCT: 7/30  SLO: 7/30  AOSLO: 7/29 | 7/31 | OCT: 8/1 | OCT: 8/3 | OCT: 8/7 | OCT: 8/14 | OCT+AOSLO: 8/21 |
| DM_190902 | F | ~4 months | SNP | 30 µL 1.2 mM | OCT: 8/3  SLO: 7/29  AOSLO: 7/31 | 8/5 | OCT: 8/6 | OCT: 8/8 | OCT: 8/12 | OCT+AOSLO: 8/19 | ND |
| DM_190901 | M | ~4 months | SNP | 30 µL 1.5 mM | OCT: 8/3  SLO: 7/30  AOSLO: 7/31 | 8/5 | OCT: 8/6 | OCT: 8/8 | OCT: 8/12 | OCT+AOSLO: 8/19 | ND |
| DM_190903 | M | ~4 months | SNP | 30 µL 1.5 mM | OCT: 7/30  SLO: 7/29  AOSLO: 7/29 | 8/13 | OCT: 8/14 | OCT: 8/16 | OCT: 8/20 | OCT+AOSLO: 8/27 | ND |
| DM_190905 | M | ~4 months | SNP | 30 µL 1.5 mM | OCT: 7/30  SLO: 7/29  AOSLO: 7/29 | 7/31 | OCT: 8/1 | OCT: 8/3 | OCT: 8/7 | OCT: 8/14 | OCT+AOSLO: 8/21 |
| DM_191202 | F | ~3.5 months | SNP | 30 µL 1.5 mM | OCT: 6/14  SLO: 6/12  AOSLO: 7/11 | 7/12 | OCT: 7/13 | OCT: 7/15 | OCT: 7/19 | ND | ND |
| DM_191203 | M | ~2.5 months | ATP | 30 µL 0.066 M | OCT: 6/14  SLO: 6/24  AOSLO: 6/22 | 6/24 | OCT: 6/25 | OCT: 6/27 | OCT: 7/1 | OCT: 7/8 | OCT+AOSLO: 7/15 |
| DM_191207 | M | ~3 months | ATP | 30 µL 0.066 M | OCT: 6/12  SLO: 6/12  AOSLO: 6/20 | 7/2 | OCT: 7/3 | OCT: 7/5 | OCT: 7/9 | OCT+AOSLO: 7/16 | ND |
| DM_191303 | M | ~2.5 months | ATP | 30 µL 0.066 M | OCT: 6/12  SLO: 6/12  AOSLO: 6/20 | 6/26 | OCT: 6/27 | OCT: 6/29 | OCT: 7/3 | ND | ND |
| DM_191204 | F | ~2.5 months | ATP | 30 µL 0.132 M | OCT: 6/12  SLO: 6/12  AOSLO: 6/21 | 6/24 | OCT: 6/25 | OCT: 6/27 | OCT: 7/1 | OCT: 7/8 | OCT+AOSLO: 7/15 |
| DM_191210 | F | ~2.5 months | ATP | 30 µL 0.132 M | OCT: 6/14  SLO: 6/12  AOSLO: 6/21 | 6/26 | OCT: 6/27 | OCT: 6/29 | OCT: 7/3 | ND | ND |
| DM_191212 | F | ~3 months | ATP | 30 µL 0.132 M | OCT: 6/14  SLO: ND  AOSLO: 6/21 | 7/2 | OCT: 7/3 | OCT: 7/5 | OCT: 7/9 | OCT+AOSLO: 7/16 | ND |
| WC_1906 | F | Unknown, of sexual maturity | IAA | 30 µL 0.5 mM | OCT: 8/16  SLO: 8/16  AOSLO: ND | 8/26 | OCT: 8/27 | OCT: 8/29 | OCT: 9/2 | OCT: 9/8 | OCT+AOSLO: 9/16 |
| WC_1901 | M | Unknown, of sexual maturity | IAA | 30 µL 1 mM | OCT: 8/16  SLO: 8/16  AOSLO: ND | 8/26 | OCT: 8/27 | OCT: 8/29 | OCT: 9/2 | OCT: 9/8 | OCT+AOSLO: 9/16 |
| MCW_190204 | F | ~2.5 months | IAA | 30 µL 3.2 mM | OCT: 8/3  SLO: 8/3  AOSLO: ND | 8/6 | OCT: 8/7 | OCT: 8/9 | OCT: 8/13 | OCT: 8/20 | ND |
| MCW_190203 | M | ~2.5 months | IAA | 30 µL 4.7 mM | OCT: 8/3  SLO: 8/3  AOSLO: ND | 8/6 | OCT: 8/7 | OCT: 8/9 | OCT: 8/13 | OCT: 8/20 | ND |
| MCW_190201 | M | ~2 months | IAA | 30 µL 7.4 mM | OCT: 7/23  SLO: 7/24  AOSLO: ND | 7/24 | OCT: 7/25 | OCT: 7/27 | OCT: 7/31 | OCT: 8/7 | OCT: 8/14 |
| MCW_190202 | F | ~2 months | IAA | 30 µL 15.4 mM | OCT: 7/23  SLO: 7/24  AOSLO: ND | 7/24 | OCT: 7/25 | OCT: 7/27 | OCT: 7/31 | OCT: 8/7 | OCT: 8/14 |

Dates are in month/day format.

All contralateral eyes received 30 µL intravitreal injections 1X PBS.

SNP, sodium nitroprusside; ATP, adenosine triphosphate; IAA, iodoacetic acid; OCT, optical coherence tomography; SLO, scanning light ophthalmoscopy; AOSLO, adaptive optics scanning light ophthalmoscopy; conc., concentration; ND, no data;
