# Supplementary material for: Chemically induced cone degeneration in the 13-lined ground squirrel
Source: Vis Neurosci. 2024 May 10;41:E002. doi: 10.1017/S0952523824000014 (PMC11106521; doi:10.1017/S0952523824000014)
Supplement: Follett et al. supplementary material 2 — Follett et al. supplementary material [file S0952523824000014sup002.docx]

Supplemental Table 2: Animal demographics, treatments, imaging dates for 2020 experiments

| Supplemental Table 2: 2020 Experiments | | | | | | | | | | | | | |
| --- | --- | --- | --- | --- | --- | --- | --- | --- | --- | --- | --- | --- | --- |
| Animal ID | Sex | Chemical | Volume & conc. | Contralateral eye | Baseline | Injection | 1 Week | 2 Weeks | 3 Weeks | 4 Weeks | 5 Weeks | 6 Weeks | 8 Weeks |
| DM_192602 | F | ATP | 20 µL 0.379M | 20 µL 0.379M | OCT: 5/22  SLO: 5/22  AOSLO: 6/5 | 7/13 | OCT+SLO*: 7/20 | OCT+SLO*: 7/27 | OCT+ SLO*: 8/4 | ND | ND | ND | OCT+SLO*+AOSLO: 9/16 (OS only) |
| DM_197913 | M | ATP | 20 µL 0.379M | 20 µL 0.379M | OCT: 5/22  SLO: 5/22  AOSLO: 5/22 | 7/13 | OCT+SLO*: 7/20 | OCT+SLO*: 7/27 | OCT+ SLO*: 8/4 | AOSLO: 8/11 (OS only) | ND | ND | OCT+SLO*+AOSLO: 9/16 (OS only) |
| MCW_190305 | M | ATP | 20 µL 0.379M | 20 µL 0.379M | OCT: 5/12; 7/10  SLO: 5/12  AOSLO: 5/13 | 7/10 | OCT+SLO*: 7/17 | OCT+SLO*: 7/24 | ND | OCT+SLO*+ AOSLO: 8/7 (OS only) | ND | ND | ND |
| DM_197911 | M | ATP | 30 µL 0.528M | None | OCT: 4/10; 4/22  SLO: 4/10  AOSLO: 4/23 | 5/18 | OCT+SLO: 5/25 | OCT+SLO: 6/1 | OCT+ SLO: 6/8 | OCT+SLO+ AOSLO: 6/15 | OCT+ SLO: 6/22 | OCT+SLO: 6/29 | OCT+SLO*+AOSLO: 7/13 |
| DM_191201 | M | ATP | 10 µL 0.723M | None | OCT: 4/13  SLO: 4/13  AOSLO: 4/30 | 6/18 | OCT+SLO: 6/25 | OCT+SLO*: 7/2 | OCT+ SLO*: 7/9 | OCT+SLO*+ AOSLO: 7/16 | OCT+ SLO*: 7/23 | OCT+ SLO*: 7/30 | OCT+SLO*+AOSLO: 8/13 |
| DM_191902 | F | ATP | 10 µL 0.723M | 10 µL 0.723M | OCT: 5/22; 7/10  SLO: 5/22  AOSLO: 6/5 | 7/10 | OCT+SLO*: 7/17 | OCT+SLO*: 7/24 | ND | OCT+SLO*+ AOSLO: 8/7 (OS only) | ND | ND | OCT+SLO*+AOSLO: 9/10 (OS only) |
| DM_198101 | M | ATP | 10 µL 0.723M | 10 µL 0.723M | OCT: 4/14; 4/22  SLO: 4/14  AOSLO: 5/7 | 7/8 | OCT+SLO*: 7/15 | OCT+SLO*: 7/22 | OCT+ SLO*: 7/29 (OD only) | OCT+SLO*+ AOSLO: 8/6 (OD only) | ND | ND | OCT+SLO*+AOSLO: 9/9 (OD only) |
| MCW_190307 | M | ATP | 10 µL 0.723M | 10 µL 0.723M | OCT: 5/10; 7/3  SLO: 5/12  AOSLO: 5/13 | 7/8 | OCT+SLO*: 7/15 | OCT+SLO*: 7/22 | OCT+ SLO*: 7/29 (OD only) | OCT+SLO*+ AOSLO: 8/6 (OD only) | ND | ND | OCT+SLO*+ AOSLO: 9/9 (OD only) |
| MCW_190308 | F | ATP | 10 µL 0.723M | None | OCT: 4/13  SLO: 4/13  AOSLO: 4/30 | 6/18 | OCT+SLO: 6/25 | OCT+SLO: 7/2 | OCT+ SLO*: 7/9 | OCT+SLO*+ AOSLO: 7/16 | OCT+SLO*: 7/23 | OCT+SLO*: 7/30 | OCT+SLO*+AOSLO: 8/13 |
| DM_198007 | M | Tgn | 30 µL 20nM | None | OCT: 4/10; 4/22  SLO: 4/10  AOSLO: 4/23 | 5/19 | OCT+SLO: 5/26 | OCT+SLO: 6/2 | OCT+ SLO: 6/9 | OCT+SLO+ AOSLO: 6/16 | OCT+SLO: 6/23 | OCT+SLO: 6/30 | OCT+SLO*+AOSLO: 7/14 |
| MCW_190309 | M | Tgn | 30 µL 20nM | None | OCT: 4/10; 4/22  SLO: 4/10  AOSLO: 4/23 | 5/19 | OCT+SLO: 5/26 | OCT+SLO: 6/2 | OCT+ SLO: 6/9 | OCT+SLO+ AOSLO: 6/16 | OCT+SLO: 6/23 | OCT+SLO: 6/30 | OCT+SLO*+AOSLO: 7/14 |
| DM_198005 | M | Tgn | 10 µL 82nM | None | OCT: 4/13  SLO: 4/13  AOSLO: 4/30 | 6/19 | OCT+SLO: 6/26 | OCT+SLO*: 7/3 | OCT+ SLO*: 7/10 | OCT+SLO*+ AOSLO: 7/17 | OCT+SLO*: 7/24 | OCT+SLO*: 7/31 | OCT+SLO*: 8/17 |
| MCW_190301 | M | Tgn | 10 µL 82nM | None | OCT: 4/13  SLO: 4/13  AOSLO: 4/30 | 6/19 | OCT+SLO: 6/26 | OCT+SLO*: 7/3 | OCT+ SLO*: 7/10 | OCT+SLO*+ AOSLO: 7/17 | OCT+SLO*: 7/24 | OCT+SLO*: 7/31 | OCT+SLO*: 8/17 |
| DM_198106 | F | Tgn | 10 µL 164nM | None | OCT: 4/14  SLO: 4/14  AOSLO: 5/7 | 7/30 | OCT+SLO*: 8/6 | OCT+SLO*: 8/14 | ND | ND | ND | ND | ND |
| MCW_190304 | F | Tgn | 10 µL 164nM | None | OCT: 4/13; 4/24  SLO: 4/6  AOSLO: 5/7 | 7/30 | OCT+SLO*:8/6 | OCT+SLO*: 8/14 | OCT+ SLO*: 8/20 | ND | ND | ND | OCT+SLO*+AOSLO: 10/6 |
| DM_198105 | M | Tm | 30 µL 0.25 µg/µL | None | OCT: 4/13; 4/24  SLO: 4/13  AOSLO: 4/29 | 5/20 | OCT+SLO: 5/27 | OCT+SLO: 6/3 | OCT+ SLO: 6/10 | OCT+SLO+ AOSLO: 6/17 | OCT+SLO: 6/24 | OCT+SLO: 7/1 | OCT+SLO*+AOSLO: 7/15 |
| MCW_190311 | M | Tm | 30 µL 0.25 µg/µL | None | OCT: 4/13; 4/24  SLO: 4/13  AOSLO: 4/29 | 5/20 | OCT+SLO: 5/27 | OCT+SLO: 6/3 | OCT+ SLO: 6/10 | OCT+SLO+ AOSLO: 6/17 | OCT+SLO: 6/24 | OCT+SLO: 7/1 | OCT+SLO*+AOSLO: 7/15 |
| DM_192002 | F | Tm | 10 µL 1.5 µg/µL | None | OCT: 5/10  SLO: 5/10  AOSLO: 5/12 | 7/15 | OCT+SLO*: 7/22 | OCT+SLO*: 7/29 | OCT+ SLO*: 8/6 | OCT+SLO*+ AOSLO: 8/12; 8/14 | ND | ND | OCT+SLO*+AOSLO: 9/10 |
| DM_197915 | M | Tm | 10 µL 1.5 µg/µL | None | OCT: 5/10  SLO: 5/10  AOSLO: 5/12 | 7/15 | OCT+SLO*: 7/22 | OCT+SLO*: 7/29 | OCT+ SLO*: 8/6 | OCT+SLO*+ AOSLO: 8/12; 8/14 | ND | ND | OCT+SLO*+AOSLO: 9/10 |
| DM_198107 | M | Tm | 10 µL 2.5 µg/µL | None | OCT: 4/14  SLO: 4/14  AOSLO: 5/7 | 9/2 | OCT+SLO*: 9/10 | OCT+SLO*: 9/16 | OCT+ SLO*: 9/22 | OCT+SLO*+ AOSLO: 10/6 | ND | ND | ND |
| MCW_190306 | F | Tm | 10 µL 2.5 µg/µL | None | OCT: 4/13  SLO: 4/14  AOSLO: 5/7 | 9/2 | OCT+SLO*: 9/10 | OCT+SLO*: 9/16 | OCT+ SLO*: 9/22 | OCT+SLO*: 10/6 | ND | ND | ND |

*Both near-infrared reflectance and short-wavelength autofluorescence SLO imaging was performed.

All animals were ≥1 year of age.

Dates are in month/day format.

ATP, adenosine triphosphate; Tgn, thapsigargin; Tm, tunicamycin; OCT, optical coherence tomography; SLO, scanning light ophthalmoscopy; AOSLO, adaptive optics scanning light ophthalmoscopy; conc., concentration; ND, no data.
