# Supplementary material for: Chemically induced cone degeneration in the 13-lined ground squirrel
Source: Vis Neurosci. 2024 May 10;41:E002. doi: 10.1017/S0952523824000014 (PMC11106521; doi:10.1017/S0952523824000014)
Supplement: Follett et al. supplementary material 3 — Follett et al. supplementary material [file S0952523824000014sup003.docx]

Supplemental Table 3: Impact of chemical and vehicle injections on choroidal thickness based on full linear mixed models and Wald tests

| Results of full choroidal thickness model and Wald tests | | | | | | | | | | |
| --- | --- | --- | --- | --- | --- | --- | --- | --- | --- | --- |
| Variables | Slope | | | Curvature | | | Cubic term | | | Overall p-value |
|  | β | | p | β | p | | β | p | |  |
| ATP | 0.0618 | **0.0022** | | -0.0066 | | **0.0137** | 0.0002 | | **0.0485** | **0.0042** |
| ATP Vehicle | 0.0768 | **0.0017** | | -0.0085 | | **0.0105** | 0.0002 | | **0.0282** | **0.0034** |
| IAA | 0.1762 | **1.42E-10** | | -0.0189 | | **1.38E-05** | 0.0005 | | **0.0015** | **8.37E-13** |
| IAA Vehicle | 0.0864 | **0.0012** | | -0.0099 | | **0.0071** | 0.0003 | | **0.0166** | **0.0024** |
| SNP | -0.0001 | 0.9962 | | -0.0018 | | 0.4710 | 0.0001 | | 0.4273 | **0.0131** |
| SNP Vehicle | -0.0122 | 0.4892 | | -0.0005 | | 0.8392 | 3.75E-05 | | 0.6799 | **0.0003** |
| Tm | 0.0340 | 0.6239 | | -0.0042 | | 0.6622 | 0.0001 | | 0.7147 | 0.7118 |
| Tgn | 0.0177 | 0.6390 | | -0.0028 | | 0.5738 | 0.0001 | | 0.5880 | 0.6261 |
